# Redox-Driven Exsolution and Dissolution Behavior of High-Entropy Spinel Catalysts: A Comparative Study of MnFeCoNiCuO_*x*_ and MnCoNiCuZnO_*x*_

**DOI:** 10.1021/acsami.5c14624

**Published:** 2025-09-02

**Authors:** Pei-Tung Chou, Cheng-Chia Kuo, Po-Yang Peng, Ying-Rui Lu, Chi-Liang Chen, Yu-Chuan Lin

**Affiliations:** † Department of Chemical Engineering, 34912 National Cheng Kung University, Tainan 70101, Taiwan; ‡ National Synchrotron Radiation Center, Hsinchu 30076, Taiwan

**Keywords:** dissolution, exsolution, high-entropy oxides, redox, reverse water−gas shift

## Abstract

High-entropy oxides (HEOs) offer tunable redox chemistry and thermal stability for catalytic applications. Here, we compare two spinel-type HEOs, MnFeCoNiCuO_*x*_ and MnCoNiCuZnO_*x*_, with similar configurational entropy but different redox behaviors under reverse water–gas shift (RWGS) conditions. Only MnFeCoNiCuO_*x*_ exhibits reversible exsolution and reincorporation of Fe/Co/Ni/Cu alloy nanoparticles (NPs) during H_2_–CO_2_ cycling, as confirmed by in situ X-ray absorption spectroscopy and wavelet-transformation. This dynamic restructuring correlates with higher concentrations of oxygen vacancy and exsolved Fe/Co/Ni/Cu alloy NPs, resulting in higher RWGS activity above 400 °C. In contrast, redox-inert Zn^2+^ in MnCoNiCuZnO_*x*_ suppresses lattice flexibility and alloy formation. These findings underscore that redox-active cations, rather than entropy alone, govern the regenerative behavior and catalytic performance in HEO systems.

## Graphical abstract

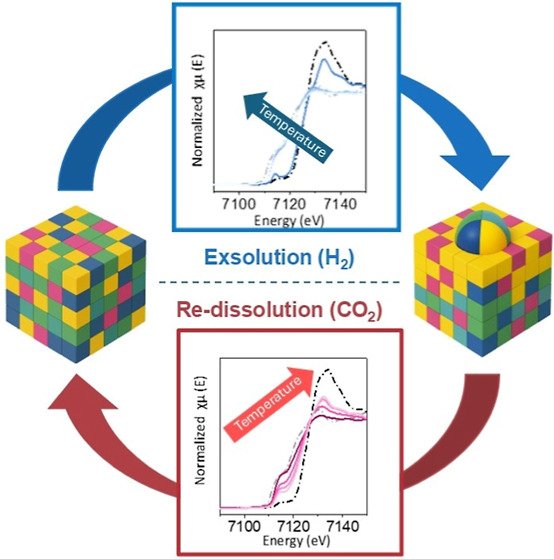

## 1 Introduction

High-entropy oxides (HEOs) are single-phase crystalline materials comprising five or more cations in near-equimolar ratios, randomly distributed over crystallographic sites.^1–3^ The resultant high configurational entropy thermodynamically stabilizes solid solutions, enabling the formation of otherwise metastable or unstable oxides.^4^ This entropy-induced stabilization also imparts structural tunability, distinctive defect chemistry, and enhanced thermal stability—attributes that position HEOs as promising catalytic platforms for reactions such as the oxygen reduction and evolution reactions.^5,6^

A key feature of HEO chemistry is the dynamic behavior of cations, enabling exsolution and redissolution in redox environments and resulting in self-regenerative structures. This phenomenon was observed in the early 2000s, when Daihatsu Motor and collaborators reported a self-regenerative catalyst—Pd-doped LaFeCoO_3_—exhibiting reversible Pd nanoparticles (NPs) migration under oxidative and reductive conditions.^7,8^ Similar behaviors were later reported with Rh- and Pt-doped perovskites, where B-site cations undergo exsolution and reincorporation.^9^ Other perovskite systems have shown comparable dynamics. For instance, the Bao group used in situ techniques such as X-ray diffraction (XRD) and scanning transmission electron microscopy to demonstrate reversible exsolution and redissolution of CoFe NPs in LaSrCoFeMoO_3_.^10,11^ Vert et al.^12^ studied the redox behavior of Ni in chromites, linking it to electrochemical performance in H_2_/CH_4_-fueled solid oxide fuel cells. Key factors enabling redissolution of exsolved NPs include substoichiometric A-site control, B-site cation selection, and tailored pre/post-treatment of perovskites.^13,14^

Configurational entropy^15^—the diversity of molecular conformations and stacking in oxides—may enable HEOs to function as self-regenerative catalysts. This is attributed to two effects: (1) structural disorder lowers Gibbs free energy, stabilizing the host lattice (support), and (2) entropy gain promotes the redissolution and oxidative reformation of NPs into their parent oxides. Zhao et al.^16^ and Hou et al.^13^ demonstrated this in the redox cycles of CO_2_ hydrogenation: the former observed Cu/Co/Ni alloy exsolution and dissolution in Co_3_MnNiCuZnO_*x*_, the latter in Co/Fe/Cu/Ni alloys from Zr_0.5_(NiFeCuMnCo)_0.5_O_*x*_. Shao et al.^17^ observed reversible cyclic evolution–dissolution of Ni, Fe, and Co to form Ni/Fe/Co alloys on halite-structured (MgCoNiMnFe)­O_*x*_ in dry reforming. Light-induced exsolution–dissolution of Co/Ni alloy in Rh-supported CoNiFeZnCrO_*x*_ in the solar-driven dry reforming of methane was revealed lately.^18^

Despite increasing interest in redox-active HEOs, the mechanisms underlying alloy formation during reduction remain insufficiently understood.^19^ It is not yet established whether HEOs with comparable configurational entropy (Δ*S*_config._) necessarily exhibit similar exsolution behavior. These open questions motivate a closer examination of how cation chemistry, rather than entropy alone, dictates the structural dynamics and catalytic function under redox cycling.

In this study, we investigate the exsolution and dissolution behavior of exsolved elements in spinel HEOs, i.e., MnFeCoNiCuO_*x*_ and MnCoNiCuZnO_*x*_, with close configurational entropies (Δ*S*_config._ = 12.4 and 12.3 J/mol/K, respectively) under reducing (H_2_) and oxidizing (CO_2_) atmospheres. Both compositions are chosen based on their successful synthesis as single-phase, entropy-stabilized spinel HEOs to provide a structurally consistent basis for comparison. The redox-driven exsolution and redissolution behavior of HEOs is dependent on the cation chemistry, even when configurational entropy is similar. The MnFeCoNiCuO_*x*_ catalyst undergoes reversible exsolution of Fe/Co/Ni/Cu alloy domains under H_2_, followed by reincorporation into the spinel lattice under CO_2_. This dynamic behavior enhances the adsorption of CO_2_ through the generation of oxygen vacancies and promotes hydrogenation via the exsolved alloy NPs, collectively leading to an increased reverse water–gas shift (RWGS) rate. In contrast, the redox-inactive Zn^2+^ in MnCoNiCuZnO_*x*_ limits structural regeneration, resulting in low RWGS activity. These findings establish that configurational entropy alone does not dictate redox dynamics or catalytic performance; instead, the rational incorporation of redox-active cations is key to unlocking the full potential of HEOs for regenerative CO_2_ catalysis.

## 2 Experimental Section

### 2.1 Materials

Nickel chloride anhydrous (98%, Sigma-Aldrich), copper chloride anhydrous (98%, SHOWA), cobalt chloride hexahydrate (98%, Sigma-Aldrich), zinc chloride anhydrous (98%, Alfa Aesar), iron chloride hexahydrate (97%, Alfa Aesar), sodium hydroxide (97%, SHOWA), and potassium permanganate (99%, SHOWA) were used as received.

### 2.2 Catalyst Synthesis

The MnCoNiCuZnO_*x*_ and MnFeCoNiCuO_*x*_ catalysts were synthesized using a solid-state ball-milling method.^16^ Briefly, 6 mmol of CoCl_2_·6H_2_O, 2 mmol of NiCl_2_, 2 mmol of CuCl_2_, and 2 mmol of FeCl_3_ (for MnFeCoNiCuO_*x*_) or 2 mmol of ZnCl_2_ (for MnCoNiCuZnO_*x*_) were added to a 45 mL zirconium oxide milling jar together with zirconium oxide milling balls with a ball-to-material ratio of 10:1. The mixture was milled in a planetary ball mill (Fritsch Pulverisette 7) at 500 rpm for 2 h. Subsequently, 24 mmol (for MnFeCoNiCuO_*x*_) or 22 mmol (for MnCoNiCuZnO_*x*_) of sodium hydroxide was added, and the mixture was milled for another 2 h. Afterward, 2 mmol of KMnO_4_ was added, followed by an additional 2 h of milling. The resulting solid was washed with deionized water, separated by centrifugation, dried overnight at 80 °C, and calcined at 600 °C for 2 h (5 °C/min) in an air stream (25 mL/min).

Some characterizations require both fresh and reduced samples, and a reduction step was conducted prior to the analysis. Each catalyst was reduced in a 20% H_2_/N_2_ stream from room temperature to 450 °C (10 °C/min) and held at 450 °C for 2 h in a fixed-bed system. The reduced HEOs were denoted as r-MnCoNiCuZnO_*x*_ and r-MnFeCoNiCuO_*x*_.

### 2.3 Characterization Methods

Catalyst composition was analyzed via inductively coupled plasma atomic emission spectroscopy (ICP-AES, Kontron S-35), and textural properties were measured by using N_2_ physisorption (Micromeritics ASAP 2020). Elemental distribution was examined by high-resolution transmission electron microscopy (JEOL JEM-2100F CS) equipped with an Oxford X-Max 20 EDS detector. Crystallinity was assessed by powder XRD (Rigaku SmartLab, Cu Kα). X-ray absorption spectra (XAS) at the Mn, Fe, Co, Ni, Cu, and Zn K-edges were collected in fluorescence mode at the TPS 32A beamline of the National Synchrotron Radiation Research Center (NSRRC). Data processing employed Athena (Demeter v0.9.26);^20^ wavelet transforms were applied to *k* ^2^-weighted XAS using a Morlet function in the *k*-range of 3–17 Å^–1^. X-ray photoelectron spectroscopy (XPS) was conducted on a PHI 5000 VersaProbe spectrometer (Al Kα, 1486.6 eV). To avoid air exposure, samples were transferred via a sealed chamber for quasi-in situ analysis. Binding energies were calibrated to the C 1s peak at 284.8 eV.

Temperature-programmed reduction (H_2_-TPR) and CO_2_ desorption (CO_2_-TPD) were performed by using an AutoChem II system (Micromeritics) with a thermal conductivity detector. Samples were prereduced at 450 °C for 2 h. After CO_2_ saturation and He purging, CO_2_-TPD was conducted under He flow (25 mL/min) at a 10 °C/min ramping rate while H_2_-TPR was conducted in a 10% H_2_/Ar stream (25 mL/min) at a 10 °C/min ramping rate.

Fourier-transform infrared spectra were recorded on a Nicolet iS50 instrument (Thermo Scientific). In situ DRIFTS experiments were performed using a Praying Mantis cell (Harrick Scientific) with CO_2_–H_2_ switching: samples were heated from 50 to 350 °C in CO_2_ (20 mL/min), then switched to H_2_ (20 mL/min) at 350 °C and held isothermally for 10 min.^21^

In situ XAS was also conducted at the NSRRC (TPS 32A). Samples were sealed in a 1.5 mm capillary, heated using a heat gun, and analyzed at various temperatures under H_2_ and CO_2_ streams to monitor Fe and Zn K-edge evolution. The Fe K-edge spectra were collected at room temperature, 350 °C, 400 °C, and 450 °C. Zn K-edge was recorded only at room temperature and 450 °C under comparable conditions.

### 2.4 Activity Evaluation

Catalytic performance was evaluated in a fixed-bed reactor system.^21,22^ Approximately 0.03 g of catalyst was loaded into quartz wool plugs. The reaction was carried out under a CO_2_/H_2_/N_2_ mixture (12.5/37.5/50.0 v/v/v) at atmospheric pressure, within a temperature range of 350–500 °C. Each activity data point reflects the average of three independent measurements; the 95% confidence interval, calculated from these replicates, is shown as the error bar. A gas hourly space velocity (GHSV) of 40,000 mL/g_cat_/h was applied, and the reactor effluent was kept at 150 °C to avoid condensation. CO_2_ conversion and product selectivity (CO and CH_4_) were calculated according to established equations.^21,22^

## 3 Results

### 3.1 Unreduced MnFeCoNiCuO_*x*_ and MnCoNiCuZnO_*x*_

Table S1 summarizes the elemental compositions determined by ICP-AES and the textural properties measured by N_2_ physisorption. The molar ratios of MnFeCoNiCuO_*x*_ and MnCoNiCuZnO_*x*_ were approximately 1:1:3:1:1 and 1:3:1:1:1, respectively, in good agreement with the target stoichiometries.^16^ By resorting to the elemental compositions, the ideal mixing formula (Δ*S*_config._ = −*R*∑_*i* = 1_^*n*^ *x*_*i*_ ln *x*_*i*_)^23^ is used to calculate the changes of configurational entropy for MnFeCoNiCuO_*x*_ (12.4 J/mol/K) and MnCoNiCuZnO_*x*_ (12.3 J/mol/K). Both HEOs exhibited comparable surface areas (46.8 and 52.6 m^2^/g) and pore volumes (0.36 and 0.28 cm^3^/g), indicating similar porous characteristics (see Figure S1).

Figure 1a,b presents the TEM-EDS elemental mapping of MnFeCoNiCuO_*x*_ and MnCoNiCuZnO_*x*_, respectively. Both samples exhibit uniform elemental dispersion, consistent with the formation of single-phase HEOs. The widespread Cu signals are partially attributed to the use of a Cu grid as the sample holder; however, the confinement of Cu signals within the particle boundary also suggests its incorporation into the HEO structure.

**Figure 1:**
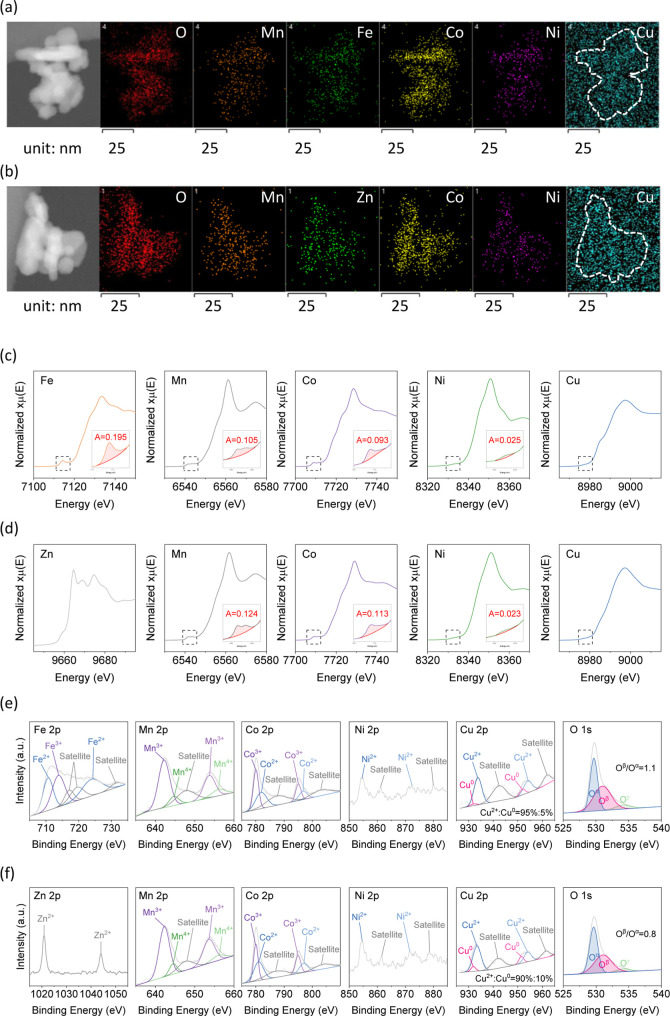
TEM images and EDS elemental mapping results of (a) MnFeCoNiCuO_*x*_ and (b) MnCoNiCuZnO_*x*_, (c) XAS K-edge spectra of Fe, Mn, Co, Ni, and Cu of MnFeCoNiCuO_*x*_, (d) XAS K-edge spectra of Zn, Mn, Co, Ni, and Cu of MnCoNiCuZnO_*x*_, (e) XPS spectra of Fe 2p, Mn 2p, Co 2p, Ni 2p, Cu 2p, and O 1s photolines of MnFeCoNiCuO_*x*_, and (f) XPS spectra of Zn 2p, Mn 2p, Co 2p, Ni 2p, Cu 2p, and O 1s photolines of MnCoNiCuZnO_*x*_.

Figure S2 shows the XRD patterns of MnFeCoNiCuO_*x*_ and MnCoNiCuZnO_*x*_. Both catalysts had diffractions at 30.6°, 36.0°, 43.7°, 57.4°, 57.7°, and 63.5°, corresponding to the (220), (311), (400), (422), (511), and (440) planes of *Fd*3̅*m* (227) spinel Co_3_O_4_ (JCPDS #80-1540).

Figure 1c,d exhibits the XAS K-edge spectra of each cation of MnFeCoNiCuO_*x*_ and MnCoNiCuZnO_*x*_. The normalized intensity of the pre-edge peak is used as an indicator of the extent of deviation from centrosymmetry: the higher the intensity, the higher the portion of cation located in the tetrahedral position.^5,24,25^ The normalized pre-edge intensity of MnFeCoNiCuO_*x*_ showed a decreasing trend as Fe (0.195) > Mn (0.105) > Co (0.093) > Ni (0.025) > Cu (0); the pre-edge intensity of MnCoNiCuZnO_*x*_: Mn (0.124) > Co (0.113) > Ni (0.023) > Cu (0). These trends suggest that both HEOs catalysts should be complex spinel structures with partial inversion.^26,27^ For MnFeCoNiCuO_*x*_, Fe cations coexist in tetrahedral and octahedral units, while the Franklinite-like Zn spectrum, which is similar to an ideal normal spinel structure,^28^ indicated that Zn cations were in tetrahedral units.^29^

Figure 1e,f shows the XPS surface analysis of MnFeCoNiCuO_*x*_ and MnCoNiCuZnO_*x*_ and Table S2 presents the relative surface composition of cations. For MnFeCoNiCuO_*x*_, the Fe 2p spectrum exhibited mixed Fe^3+^ (55%) and Fe^2+^ (45%). The Mn 2p spectrum revealed Mn^4+^ and Mn^3+^ at 14% and 86%, Co 2p showed Co^3+^ (54%) and Co^2+^ (46%), Ni appeared as Ni^2+^ (100%), and Cu appeared as Cu^2+^ (95%) and Cu^0^ (5%). For MnCoNiCuZnO_*x*_, Zn 2p showed predominantly Zn^2+^. Mn^4+^ (13%) and Mn^3+^ (87%), Co^3+^ (48%) and Co^2+^ (52%), Ni^2+^ (100%), Cu^2+^ (90%), and Cu^0^ (10%) were observed. The O 1s spectra of both catalysts showed three components: lattice oxygen (O^α^, 529.4 eV), oxygen in surface vacancies (O^β^, 531.0 eV), and hydroxyl/surface-adsorbed oxygen (O^γ^, 534.0 eV).^30,31^ The O^β^/O^α^ ratio was higher in MnFeCoNiCuO_*x*_ (1.1) than in MnCoNiCuZnO_*x*_ (0.8), indicating a greater concentration of the surface oxygen vacancy (O_v_) of the former.

Figure 2a exhibits the CO_2_-TPD profiles of MnFeCoNiCuO_*x*_ and MnCoNiCuZnO_*x*_. Both catalysts had similar CO_2_ desorption profiles, peaked at low- (ca. 125 °C) and medium- (ca. 180–190 °C) regions. The amounts of desorbed CO_2_ at low- and medium-temperatures were 121.3 and 135.7 μmol/g for MnFeCoNiCuO_*x*_ and 135.8 and 143.6 μmol/g for MnCoNiCuZnO_*x*_, respectively. MnCoNiCuZnO_*x*_ showed a slightly higher CO_2_ desorption amount (279.4 μmol/g) than that of MnFeCoNiCuO_*x*_ (257.0 μmol/g).

**Figure 2:**
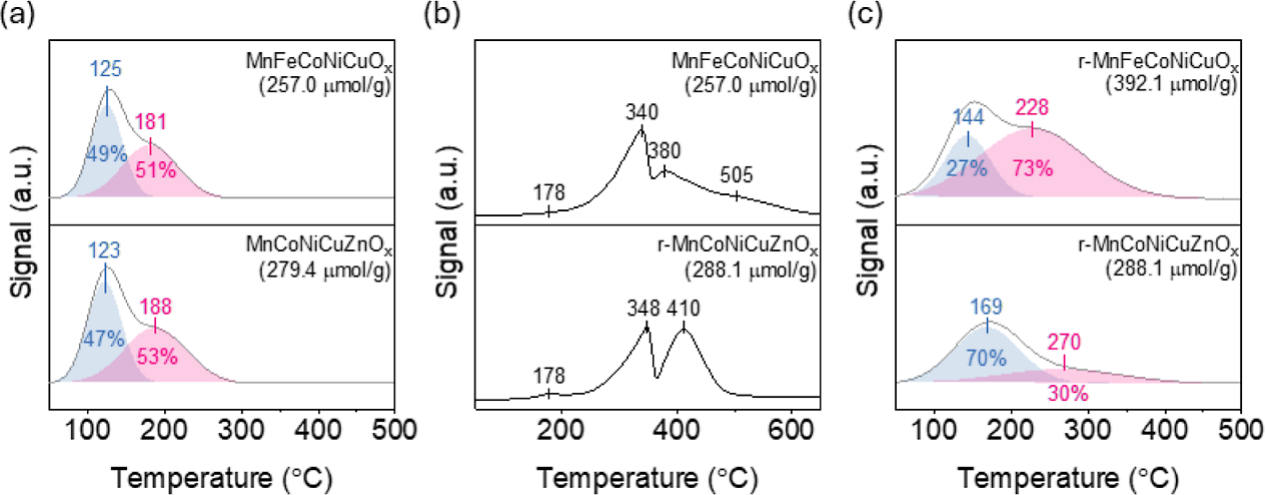
(a) CO_2_-TPD profiles of MnFeCoNiCuO_*x*_ and MnCoNiCuZnO_*x*_, (b) H_2_-TPR profiles of MnFeCoNiCuO_*x*_ and MnCoNiCuZnO_*x*_, and (c) CO_2_-TPD profiles of r-MnFeCoNiCuO_*x*_ and r-MnCoNiCuZnO_*x*_. Conditions: CO_2_-TPD was recorded in a He stream (25 mL/min) at a ramp rate of 10 °C/min; H_2_-TPR, 10% H_2_/Ar (25 mL/min) at a ramp rate of 10 °C/min.

Figure 2b shows the H_2_-TPR profiles of MnFeCoNiCuO_*x*_ and MnCoNiCuZnO_*x*_. Both catalysts exhibit multiple reduction peaks: a minor shoulder at 178 °C (Cu^2+^ → Cu^0^),^32^ followed by overlapping peaks at 340–350 °C (Ni^2+^ → Ni^0^)^32^ and 380–410 °C (Co cations reduction).^33,34^ An additional shoulder at 505 °C appears only for MnFeCoNiCuO_*x*_, corresponding to Fe^3+^ reduction.^17^ Total H_2_ consumption was 10.5 mmol/g for MnFeCoNiCuO_*x*_ and 9.1 mmol/g for MnCoNiCuZnO_*x*_. The 1.4 mmol/g difference is attributed to the presence of reducible Fe^3+^ in the former, whereas Zn^2+^ in the latter seemed to be unreducible below 800 °C.

### 3.2 Reduced MnFeCoNiCuO_*x*_ and MnCoNiCuZnO_*x*_

Figure 3a,b shows representative TEM images and EDS mappings of r-MnFeCoNiCuO_*x*_ and r-MnCoNiCuZnO_*x*_ NPs, respectively. Compared with the fresh forms, these reduced particles exhibit uneven elemental distributions, particularly in the highlighted red-boxed areas, consistent with localized exsolution sites. For r-MnFeCoNiCuO_*x*_, O and Mn were nearly undetectable in the highlighted region, while for r-MnCoNiCuZnO_*x*_, O, Mn, and Zn were nearly unseen. This implied that the reduction treatment induced the relocation of some elements. That is, during the reduction, Fe, Co, Ni, and Cu can be redispersed in r-MnFeCoNiCuO_*x*_ while Co, Ni, and Cu can be relocated in r-MnCoNiCuZnO_*x*_.

**Figure 3:**
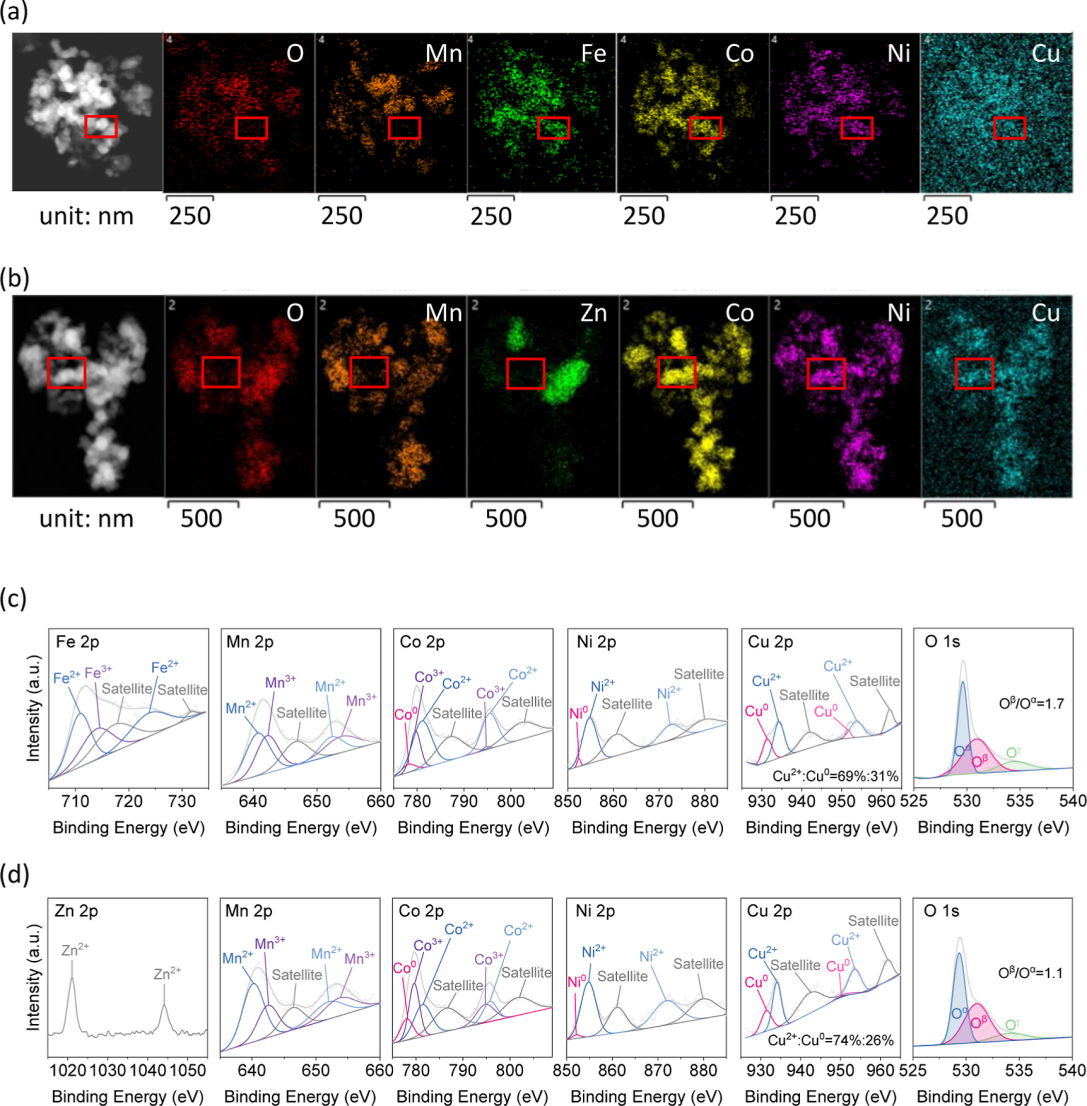
TEM images and EDS elemental mapping results of (a) r-MnFeCoNiCuO_*x*_ and (b) r-MnCoNiCuZnO_*x*_, (c) XPS spectra of Fe 2p, Mn 2p, Co 2p, Ni 2p, Cu 2p, and O 1s photolines of r-MnFeCoNiCuO_*x*_, and (d) XPS spectra of Zn 2p, Mn 2p, Co 2p, Ni 2p, Cu 2p, and O 1s photolines of r-MnCoNiCuZnO_*x*_.

Figure S2 displays the XRD patterns of r-MnFeCoNiCuO_*x*_ and r-MnCoNiCuZnO_*x*_. Both reduced catalysts exhibit a doublet near 44.0°, along with several minor diffraction peaks, suggesting the formation of Co/Ni/Cu-based alloy phases.^35^

Figure 3c,d shows the XPS spectra of r-MnFeCoNiCuO_*x*_ and r-MnCoNiCuZnO_*x*_ and Table S2 summarizes the relative surface cation compositions. The spectrum of each element was like its unreduced counterpart, with a decreased ratio of high-to-low cation/metal oxidation state. For r-MnFeCoNiCuO_*x*_, the Fe 2p spectrum exhibited Fe^3+^ (46%) and Fe^2+^ (54%). The Mn 2p spectrum showed Mn^3+^ (45%) and Mn^2+^ (55%), Co 2p showed Co^3+^ (28%), Co^2+^ (64%), and Co^0^ (8%), Ni showed Ni^2+^ (96%) and Ni^0^ (4%), and Cu showed Cu^2+^ (64%) and Cu^0^ (36%). For r-MnCoNiCuZnO_*x*_, the Zn 2p spectrum was nearly identical to its unreduced form, showing a characteristic Zn^2+^ photoline. The other cations showed compositions of Mn^3+^ (34%) and Mn^2+^ (66%), Co^3+^ (41%), Co^2+^ (40%) and Co^0^ (19%), Ni^2+^ (97%) and Ni^0^ (3%), and Cu^2+^ (65%) and Cu^0^ (35%).

The O^β^/O^α^ ratio of r-MnFeCoNiCuO_*x*_ (1.7) was higher than that of r-MnCoNiCuZnO_*x*_ (1.1), and both values were higher than those of the pristine forms. That is, the reduction of HEOs develops the corresponding O_v_ sites, and r-MnFeCoNiCuO_*x*_ showed a higher relative O_v_ composition than that of r-MnCoNiCuZnO_*x*_.

Figure 2c shows the CO_2_-TPD profiles of r-MnFeCoNiCuO_*x*_ and r-MnCoNiCuZnO_*x*_. The low- and medium-temperature desorption peaks shifted from ca. 125 to 140–170 °C and from ca. 180–190 to 230–270 °C, respectively, compared to their unreduced counterparts (see Figure 2a). The total amount of desorbed CO_2_ from the low- and medium-temperature desorption peaks increased from 257.0 to 392.1 μmol/g for r-MnFeCoNiCuO_*x*_ and from 279.4 to 288.1 μmol/g for r-MnCoNiCuZnO_*x*_. This corresponds to an enhancement of 135.1 μmol/g in the CO_2_ adsorption capacity for r-MnFeCoNiCuO_*x*_, more than an order higher than the increase observed for r-MnCoNiCuZnO_*x*_ (8.7 μmol/g).

To further understand the redispersion/exsolution behaviors of r-MnFeCoNiCuO_*x*_ and r-MnCoNiCuZnO_*x*_, the wavelet-transformed XAS (WT-XAS) analysis of their reduced forms was conducted, shown in Figure 4. The WT-XAS 2D contour plots of Co, Ni, Cu, and Fe foils and ZnO were included for comparison. The WT-XAS 2D contour plot exhibits not only the interatomic distance (*R* space) but also backscattering amplitude (*k* space), allowing the identification of multiple bonds having similar bond distances.^36,37^

**Figure 4:**
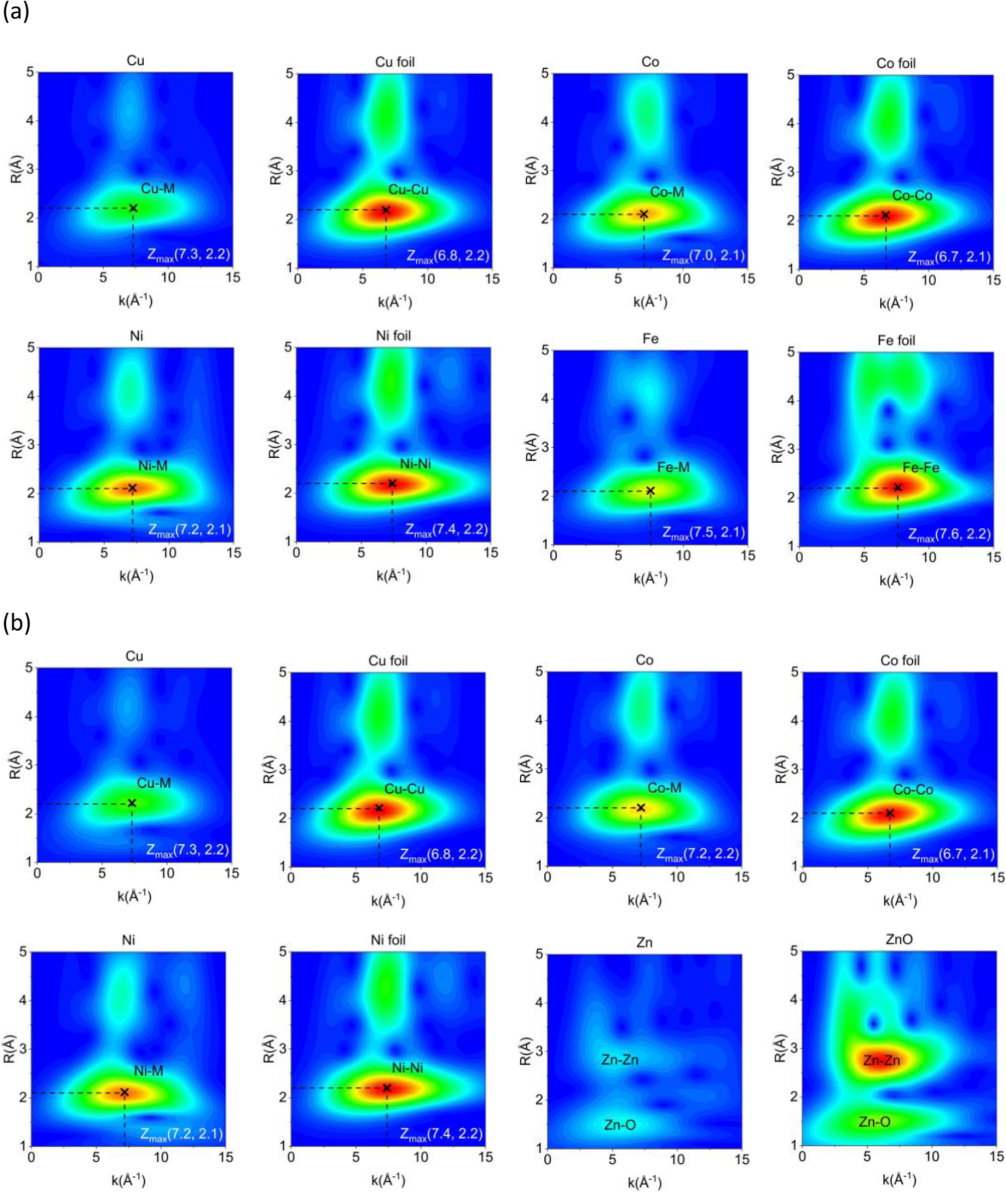
(a) Wavelet-transformed XAS (WT-XAS) of Cu, Co, Ni, and Fe K-edge spectra of r-MnFeCoNiCuO_*x*_ and (b) Cu, Co, Ni, and Zn K-edge spectra of r-MnCoNiCuZnO_*x*_. The references including metallic foils of Cu, Co, Ni, Fe, and ZnO were included.

For r-MnFeCoNiCuO_*x*_, the peaks of Fe–M (*R* ≈2.1 Å and *k* ≈7.5 cm^–1^), Co–M (*R* ≈2.1 Å and *k* ≈7.0 cm^–1^), Ni–M (*R* ≈2.1 Å and *k* ≈7.2 cm^–1^), and Cu–M (*R* ≈2.2 Å and *k* ≈7.3 cm^–1^) (M = metallic Fe, Co, Ni, and Cu) were close to their respective references, i.e., Fe–Fe (*R* ≈2.2 Å and *k* ≈7.6 cm^–1^), Co–Co (*R* ≈2.1 Å and *k* ≈6.7 cm^–1^), Ni–Ni (*R* ≈2.2 Å and *k* ≈7.4 cm^–1^), and Cu–Cu (*R* ≈2.2 Å and *k* ≈6.8 cm^–1^).^38^ These slight deviations in *R* and *k* values underlined that the exsolved Fe, Co, Ni, and Cu exist in alloyed rather than pure elemental states. The same could be stated for r-MnCoNiCuZnO_*x*_ besides Zn, which possessed an oxide form like the pattern of ZnO.

### 3.3 Activity Test

Figure 5a exhibits the RWGS activity test at 350–500 °C. CO_2_ conversions increased from 20.1% ± 3.0% to 43.5% ± 2.8% for MnFeCoNiCuO_*x*_ and 13.9% ± 2.8% to 38.3% ± 2.4% for MnCoNiCuZnO_*x*_. CO dominated (>96%) in the tested temperatures. In 400 °C, CH_4_ (3.9% and 2.0%) was detected for MnCoNiCuZnO_*x*_ and MnFeCoNiCuO_*x*_ while no CH_4_ was detected above 450 °C for both catalysts. A close inspection can find a jump of CO_2_ conversion in 400 (27.4%)–450 °C (39.6%) for MnFeCoNiCuO_*x*_. Pure ZnO was tested under RWGS conditions and found inactive. Figure 5b shows the 100 h durability test for both catalysts at 450 °C. The conversions (MnFeCoNiCuO_*x*_ = 40.0% and MnCoNiCuZnO_*x*_ = 29.2%) were nearly unchanged with only CO as the detectable product.

**Figure 5:**
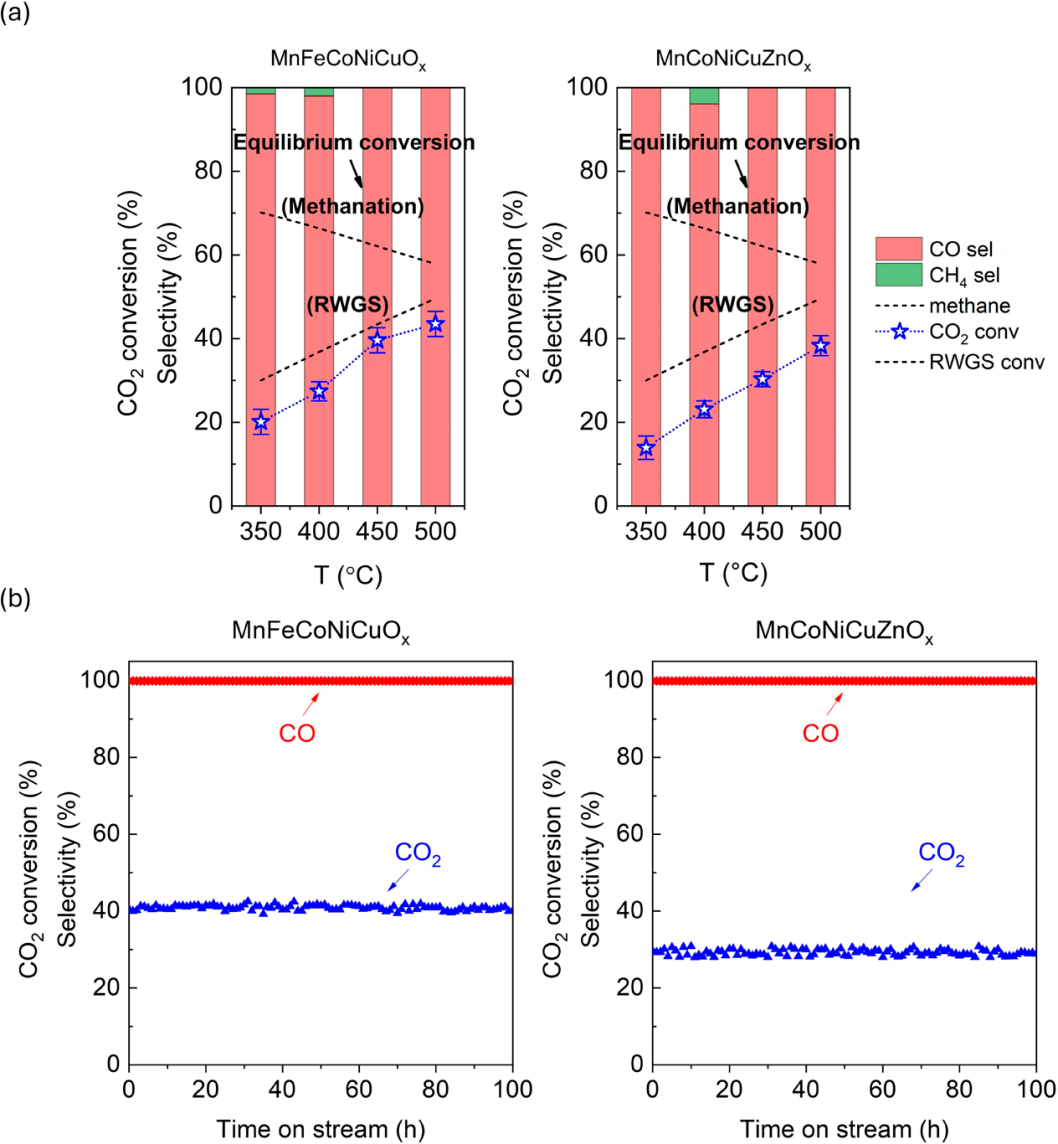
(a) Activity evaluation in 350–500 °C and (b) 100 h durability test for MnFeCoNiCuO_*x*_ and MnCoNiCuZnO_*x*_ at 450 °C. Reaction conditions: *P* = 0.1 MPa, H_2_/CO_2_ = 3, and GHSV = 40,000 mL/g_cat_/h.

Figure S3 presents the in situ DRIFTS results under CO_2_–H_2_ switching. In the CO_2_ stream, both catalysts exhibited characteristic bands at 1590 and 1300 cm^–1^ (Δν ≈290 cm^–1^), along with a minor band at 1040 cm^–1^, attributable to bidentate chelating carbonate (b-*CO_3_).^39,40^ These b-*CO_3_ bands intensified with increasing temperature from 50 to 350 °C. Upon switching to H_2_ at 350 °C, the b-*CO_3_ bands progressively diminished over time, accompanied by the appearance of gaseous CO bands at 2176 and 2113 cm^–1^. Simultaneously, bands at 1540 and 1340 cm^–1^ with a reduced Δν of ∼200 cm^–1^ indicated the presence of residual b-*CO_3_ due to varying basicity on the catalyst surface, and the new band at 1475 cm^–1^ indicated the formation of surface bicarbonate (*HCO_3_), likely due to partial reduction of b-*CO_3_ in H_2_.^39^

### 3.4 Redox Property Evaluation

To investigate exsolution behavior, in situ XAS measurements were performed on the Fe K-edge of MnFeCoNiCuO_*x*_ and the Zn K-edge of MnCoNiCuZnO_*x*_ during H_2_–CO_2_ switching tests (see Figure 6).

**Figure 6:**
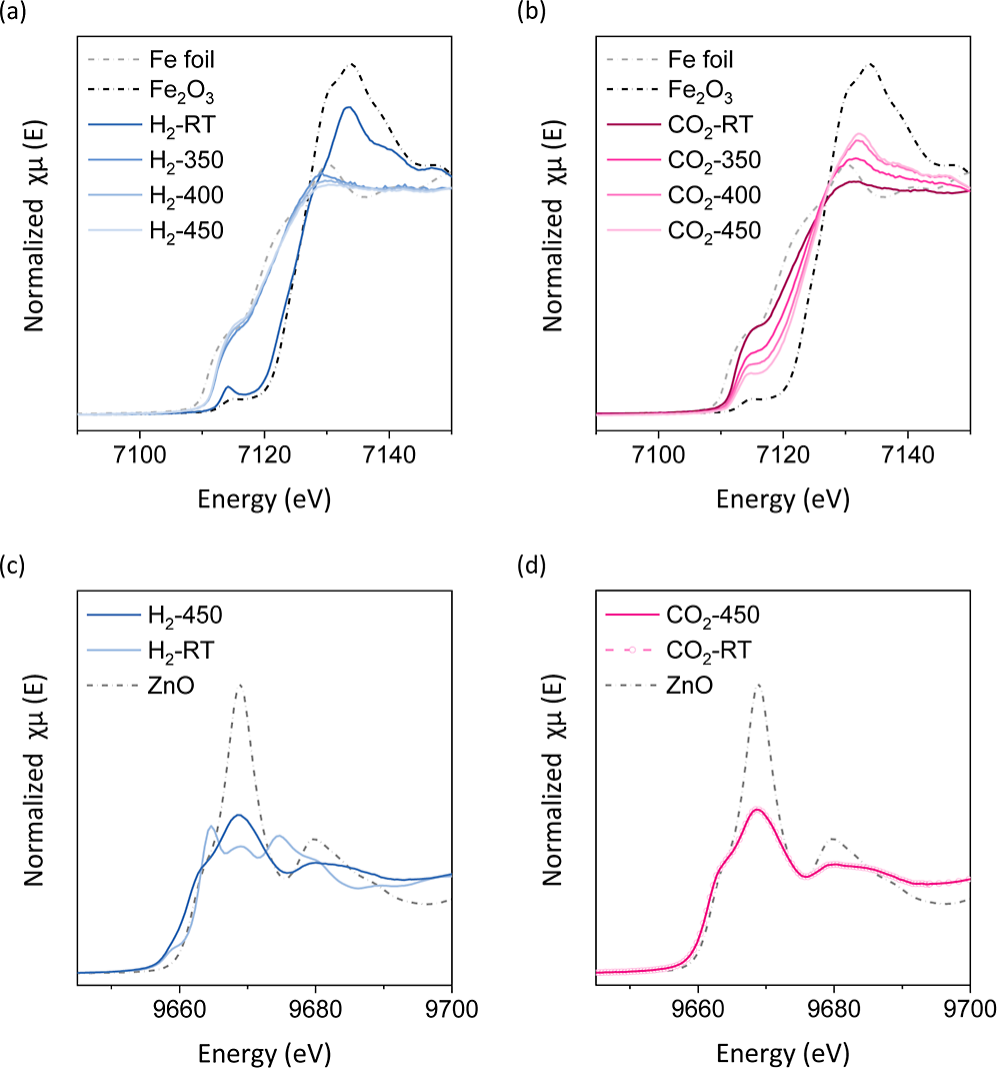
In situ XAS spectra of Fe K-edge of MnFeCoNiCuO_*x*_ in (a) H_2_ and (b) CO_2_ at room temperature and 350, 450, and 500 °C and (b) Zn K-edge of MnCoNiCuZnO_*x*_ at room temperature and 450 °C in (c) H_2_ and (d) CO_2_.

For MnFeCoNiCuO_*x*_ under H_2_ at room temperature, the Fe K-edge spectrum exhibited a strong white line with an edge at 7126.1 eV. As the temperature increased to 350–450 °C, both the white line intensity and edge position decreased, shifting to 7112.1 eV. Linear combination analysis indicated a reduction in the Fe oxidation state from +2.7 to +0.2 (see Figure S4a). Upon exposure to CO_2_, the Fe edge showed the reverse trend: an increase in white line intensity and an upward shift in edge energy (from 7112.1 eV at room temperature to 7126.1 eV at 450 °C), with the Fe oxidation state increasing from +0.2 to +2.7 (see Figure S4b). These results demonstrate a reversible redox process of Fe—exsolution in H_2_ and redissolution in CO_2_—characteristic of MnFeCoNiCuO_*x*_.

In contrast, at 450 °C under H_2_, the Zn K-edge spectrum of r-MnCoNiCuZnO_*x*_ evolves to closely match that of ZnO, confirming the absence of Zn–metal bonding and demonstrating that Zn^2+^ remains excluded from the exsolved alloy NPs. However, upon switching to CO_2_ at 450 °C, the spectrum remained unchanged, indicating that Zn^2+^ does not reincorporate into the spinel units of MnCoNiCuZnO_*x*_. This confirms the redox-inactive nature of Zn in MnCoNiCuZnO_*x*_ under the tested conditions.

Figure S2 further confirms the exsolution in H_2_ and redissolution in CO_2_ through XRD analysis. The CO_2_ reoxidized r-MnFeCoNiCuO_*x*_ and r-MnCoNiCuZnO_*x*_ exhibited similar spinel diffraction patterns to their pristine forms, albeit with reduced diffraction peak intensities.

## 4 Discussion

### 4.1 Redox-Driven Structural Dynamics

This study highlights the critical role of cation identity in dictating redox-responsive exsolution within spinel-type HEOs under RWGS conditions. By examining MnFeCoNiCuO_*x*_ and MnCoNiCuZnO_*x*_, both successfully synthesized as single-phase, entropy-stabilized spinels containing five cations—we maintain nearly identical configurational entropy (Δ*S*_config._ = 12.4 and 12.3 J/mol/K, respectively), enabling an assessment of how the substitution of redox-active Fe^3+^ versus redox-inert Zn^2+^ alters redox dynamics and catalytic behaviors.

In situ XAS and WT-XAS analyses confirm that MnFeCoNiCuO_*x*_ undergoes reversible exsolution and redissolution of Fe/Co/N/Cu NPs alloy under H_2_–CO_2_ cycles. In contrast, MnCoNiCuZnO_*x*_ shows limited redox responsiveness, with Zn^2+^ resisting the redox transformation. A possible explanation of Zn’s redox inertness may be due to its high standard Gibbs free energy of reduction (Δ*G*° > 50 kJ/mol).^41^ Figure 7 illustrates the redox dynamics of MnFeCoNiCuO_*x*_ and MnCoNiCuZnO_*x*_.

**Figure 7:**
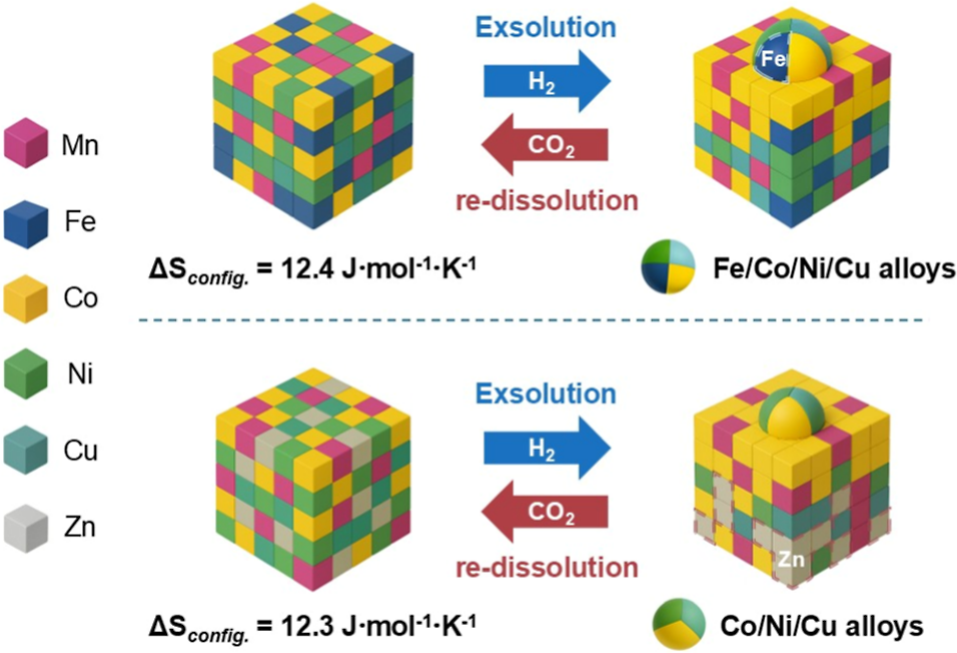
The scheme of reversible exsolution and redissolution of Fe/Co/Ni/Cu NPs alloy on MnFeCoNiCuO_*x*_ and Co/Ni/Cu NPs alloy on MnCoNiCuZnO_*x*_ with the change of the local coordination of Zn^2+^.

### 4.2 Surface Oxygen Vacancies and RWGS Activity

The enhanced catalytic performance of MnFeCoNiCuO_*x*_ correlates with its higher surface O_ν_ concentration, as indicated by an increased O^β^/O^α^ ratio and greater H_2_ uptake relative to MnCoNiCuZnO_*x*_. Oxygen vacancies play a role in facilitating CO_2_ activation, consistent with previous findings in other HEOs such as Zr_0.5_(NiFeCuMnCo)_0.5_O_*x*_ ^13^ and CoNiFeZnCrO_*x*_.^18^ In our system, the onset of high RWGS activity above 400 °C in MnFeCoNiCuO_*x*_ is accompanied by the formation of Fe/Co/Ni/Cu alloy NPs, mirroring trends observed in La­(FeCoNiCrMn)­O_3_ perovskites^42^ and (CrMnFeCoNiCuZn)_3_O_4_ spinels,^43^ where exsolved NPs and elevated O_ν_ concentrations lower light-off temperatures in CO oxidation. Furthermore, the high CO selectivity and durability of MnFeCoNiCuO_*x*_ under moderate conditions underscores its industrial promise of RWGS, especially in processes coupling renewable hydrogen with CO_2_ to produce syngas for downstream value-added products.

Complementary CO_2_-TPD and in situ DRIFTS analyses further support these observations. MnFeCoNiCuO_*x*_ exhibits greater CO_2_ adsorption capacity in the reduced state, with bidentate carbonate (b-*CO_3_) identified as the dominant intermediate formed via chemisorption at O_ν_ sites.^44,45^ Under H_2_ flow, the transient intensification and subsequent decline of b-*CO_3_ signals suggest a RWGS mechanism involving carbonate formation followed by hydrogenation—likely catalyzed by the exsolved Fe/Co/Ni/Cu NPs.^21,46^

### 4.3 Implications for Entropy-Stabilized Catalyst Design

While configurational entropy supports phase stability in HEOs, it does not guarantee redox adaptability or catalytic regeneration. The presence of redox-flexible cations, such as Fe^3+^, is essential for enabling dynamic structural evolution and reversible alloy formation. In contrast, redox-inert Zn^2+^ dilutes active sites and is inactive in CO_2_ activation, consistent with previous reports of its inactivity in standalone catalytic conversion.^47,48^ Thus, effective catalyst design should integrate entropy stabilization with the selection of redox-active components to tailor structural dynamics and reaction performance.

Spinel-type HEOs have proven their value in real-world catalysis, offering exceptional thermal stability, resistance to deactivation (e.g., anticoking and antisintering), and dynamic active-site behavior under harsh conditions. For example, a (CoCrFeNiAl)_3_O_4_ spinel HEO achieved over 80% H_2_ yield with sustained performance in ethanol steam reforming at 600 °C, likely due to the self-reorganization and reversible metal exsolution.^49^ This underlines the structural resilience and redox agility required in industrial catalysts, reinforcing the practical relevance of our entropy-stabilized, redox-tunable HEOs.

## 5 Conclusions

This work reveals the important role of redox-active cation selection in enabling reversible exsolution–dissolution behavior in high-entropy spinel oxides for RWGS catalysis. MnFeCoNiCuO_*x*_ demonstrates dynamic redox adaptability through the reversible formation and redissolution of Fe/Co/Ni/Cu alloy NPs under H_2_–CO_2_ cycling—behavior absent in the Zn-containing MnCoNiCuZnO_*x*_. The enhanced RWGS activity of MnFeCoNiCuO_*x*_, particularly above 400 °C, is attributed to the synergy of oxygen vacancies and exsolved Fe/Co/Ni/Cu alloy NPs. Notably, a similar configurational entropy in both systems does not guarantee redox responsiveness; rather, the presence of reducible cations is essential for catalytic regeneration. These findings offer a rational strategy for designing advanced HEOs catalysts by coupling entropy stabilization with targeted redox-active cation engineering.

## Supplementary Material



## Data Availability

Data will be made available on request.

## Notes

N_2_ isotherms and porosities; XPS-estimated relative surface cation composition; XRD patterns of MnFeCoNiCuO_*x*_, r-MnFeCoNiCuO_*x*_, and reoxidized r-MnFeCoNiCuO_*x*_; MnCoNiCuZnO_*x*_, r-MnCoNiCuZnO_*x*_, and reoxidized r-MnCoNiCuZnO_*x*_; in situ DRIFT analysis of a CO_2_–H_2_ switching test; and first derivatives of Fe XAS K-edge spectra of MnFeCoNiCuO_*x*_ in H_2_ and CO_2_ streams (PDF)
