# Supplementary material for: Redox-Driven Exsolution and Dissolution Behavior of High-Entropy Spinel Catalysts: A Comparative Study of MnFeCoNiCuO x and MnCoNiCuZnO x
Source: ACS Appl Mater Interfaces. 2025 Sep 2;17(37):52315–24. doi: 10.1021/acsami.5c14624 (PMC12447387; doi:10.1021/acsami.5c14624)
Supplement: Supplementary file 1 [file am5c14624_si_001.pdf]

Supporting Information

for

Redox-Driven Exsolution and Dissolution Behavior of High Entropy Spinel Catalysts: A Comparative Study of $MnFeCoNiCuO_x$ and $MnCoNiCuZnO_x$

Pei-Tung Chou,[a] Cheng-Chia Kuo,[a] Po-Yang Peng,[b] Ying-Rui Lu,[b] Chi-Liang Chen,[b] and Yu-Chuan Lin[a,*]

[a] Department of Chemical Engineering, National Cheng Kung University, Tainan 70101, Taiwan

[b] National Synchrotron Radiation Center, Hsinchu 30076, Taiwan

*Corresponding author's Email: yclin768@mail.ncku.edu.tw (Y.-C. Lin)

Number of pages: 7

Number of tables: 2

Number of figures: 4

Table S1. Elemental composition estimated by ICP-AES and porosity obtained by N_2 physisoprtion analysis.

Catalyst	Mn	Fe	Co	Ni	Cu	Zn	S_{BET}	V_{total}
			(wt%)				(m^2/g)	(cm^3/g)
$MnFeCoNiCuO_x$	9.4	9.5	28.1	9.5	9.3	X	46.8	0.36
$MnCoNiCuZnO_x$	9.4	X	26.0	9.6	9.7	9.8	52.6	0.28

Table S2. XPS-estimated surface compositions of tested HEOs and their reduced forms.

Sample	Oxidation state	Mn	Fe	Co	Ni	Cu	Zn
				(%)			
$MnFeCoNiCuO_x$	0	-	-	-	-	5	N.A.
	2+	-	45	46	100	95	
	3+	86	55	54	-	-	
	4+	14	-	-	-	-	
r-$MnFeCoNiCuO_x$	0	-	-	8	4	36	N.A.
	2+	55	54	64	96	64	
	3+	45	46	28	-	-	
$MnCoNiCuZnO_x$	0	-	N.A.	-	-	10	-
	2+	-		52	100	90	100
	3+	87		48	-	-	-
	4+	13		-	-	-	-
r-$MnCoNiCuZnO_x$	0	-	N.A.	19	3	35	-
	2+	66		40	97	65	100
	3+	34		41	-	-	-

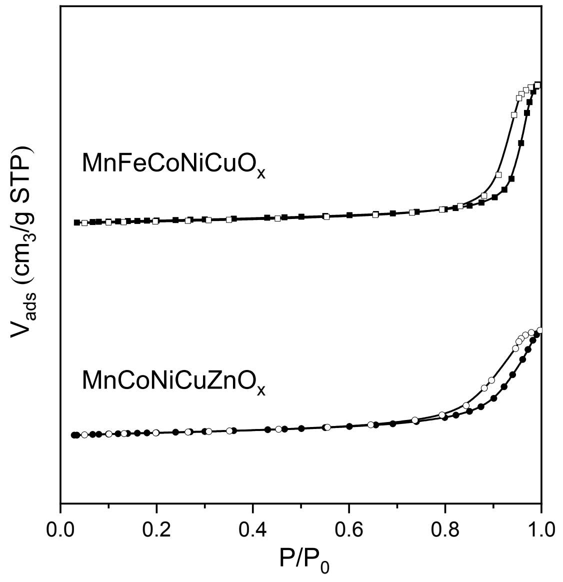

Figure S1. N_2 adsorption-desorption isotherms of $MnFeCoNiCuO_x$ and $MnCoNiCuZnO_x$.

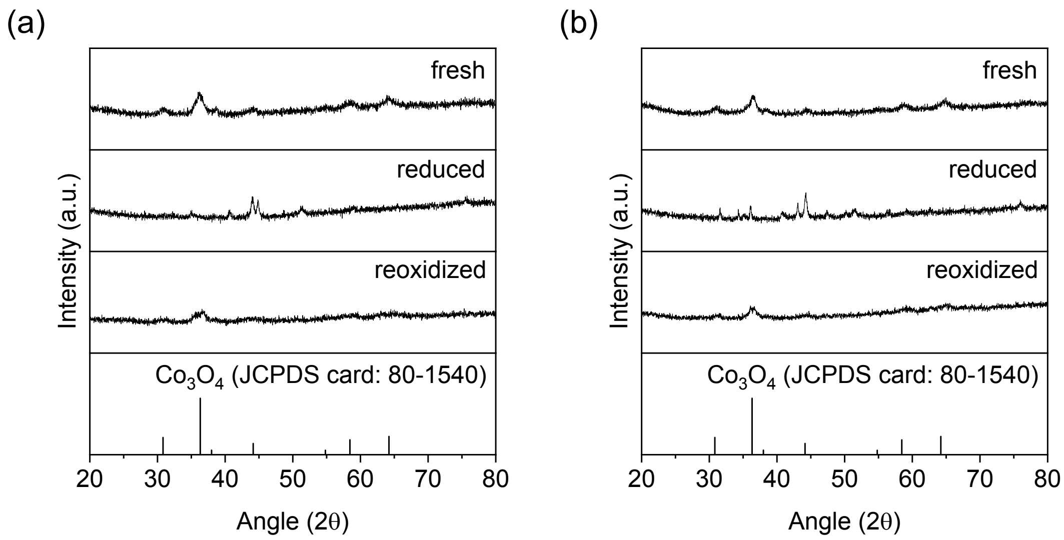

Figure S2. XRD patterns of (a) fresh $MnFeCoNiCuO_x$, r-$MnFeCoNiCuO_x$, and CO_2 re-oxidized r-$MnFeCoNiCuO_x$ and (b) fresh $MnCoNiCuZnO_x$, r-$MnCoNiCuZnO_x$, and CO_2 re-oxidized r-$MnCoNiCuZnO_x$.

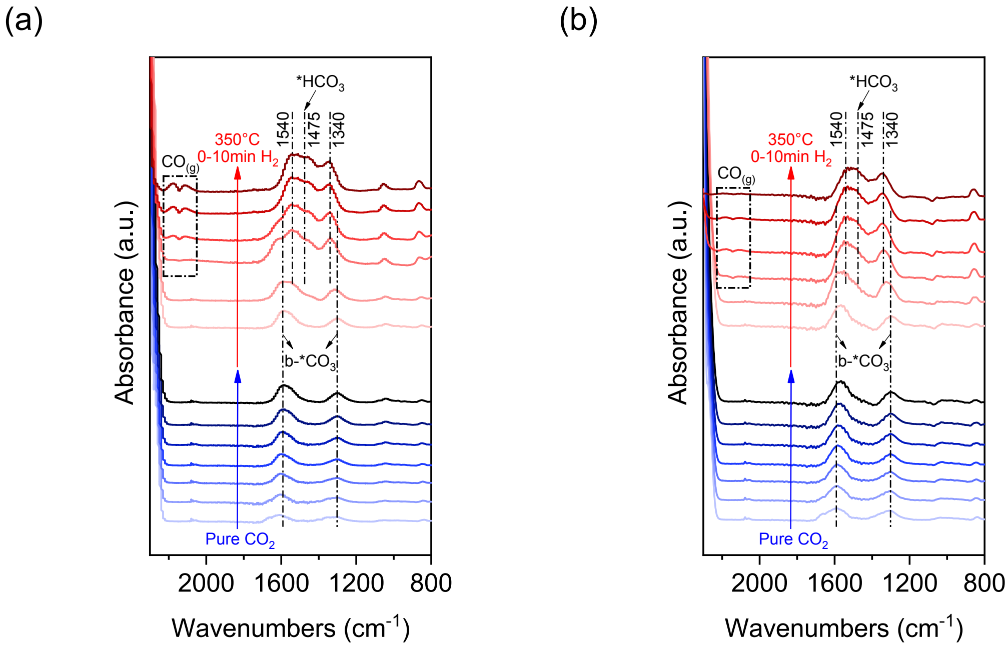

Figure S3. In-situ DRIFT analysis of a CO_2–H_2 switching test for (a) $MnFeCoNiCuO_x$ and (b) $MnCoNiCuZnO_x$

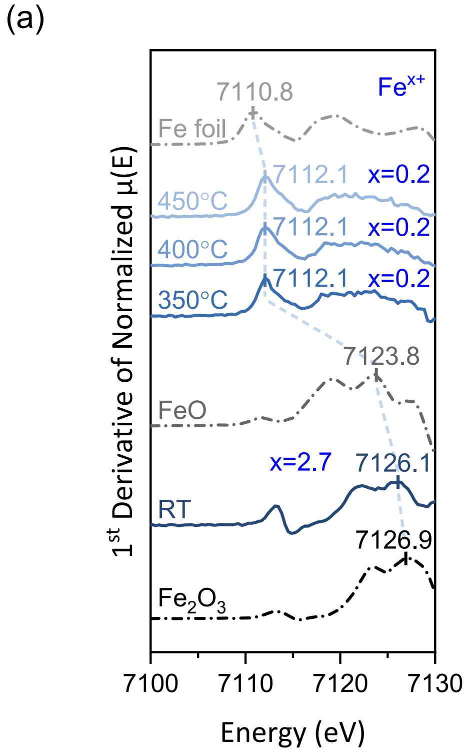

Figure S4. The first derivatives of Fe XANES K-edge spectra of MnFeCoNiCuO$_x$ in (a) H_2 and (b) CO_2 at room temperature, 350, 450, and 500 °C. Linear combination was used to obtain the valance state of Fe by using Fe foil, FeO and Fe_2O_3 as the references.
